# Photosynthetic Responses, Growth, Production, and Tolerance of Traditional Varieties of Cowpea under Salt Stress

**DOI:** 10.3390/plants11141863

**Published:** 2022-07-18

**Authors:** Saulo Samuel Carneiro Praxedes, Miguel Ferreira Neto, Aline Torquato Loiola, Fernanda Jessica Queiroz Santos, Bianca Fernandes Umbelino, Luderlândio de Andrade Silva, Rômulo Carantino Lucena Moreira, Alberto Soares de Melo, Claudivan Feitosa de Lacerda, Pedro Dantas Fernandes, Nildo da Silva Dias, Francisco Vanies da Silva Sá

**Affiliations:** 1 Department of Agronomic and Forest Sciences, Federal Rural University of the Semi-Arid—UFERSA, Mossoró 59625-900, Brazil; saulosamuel@rn.gov.br (S.S.C.P.); ninator4@gmail.com (A.T.L.); fernanda.santos76421@alunos.ufersa.edu.br (F.J.Q.S.); bianca.umbelino@alunos.ufersa.edu.br (B.F.U.); nildo@ufersa.edu.br (N.d.S.D.); 2 Center of Technology and Natural Resources, Federal University of Campina Grande—UFCG, Campina Grande 58428-830, Brazil; luderlandioandrade@gmail.com (L.d.A.S.); romulocarantino@gmail.com (R.C.L.M.); pedrodantasfernandes@gmail.com (P.D.F.); 3 Department of Biology, Universidade Estadual da Paraíba, Campina Grande 58429-500, Brazil; alberto@uepb.edu.br; 4 Department of Agricultural Engineering, Federal University of Ceará, Fortaleza 60440-593, Brazil; cfeitosa@ufc.br

**Keywords:** *Vigna unguiculata* (L.) Walp., salinity, gas exchange, photochemical efficiency, photochemical quenching, yield

## Abstract

**Highlights:**

- Salinity tolerance among traditional varieties of cowpea is variable.
- High net photosynthesis is observed in cowpea-tolerant varieties under salt stress.
- The decrease in photosynthesis is caused by stomatal restriction and low photochemical efficiency.
- Cowpea varieties are similar in photochemical efficiency and photochemical quenching.
- The decrease in pod number and seeds per pod are the main effects of salinity on cowpea production.

**Abstract:**

Cowpea is the main subsistence crop—protein source—for the Brazilian semi-arid region. The use of salt-stress-tolerant varieties can improve crop yields. We evaluated the effect of irrigation with brackish water on the growth, photosynthetic responses, production, and tolerance of fifteen traditional varieties of cowpea. The experiment was conducted in randomized blocks, in a 15 × 2 factorial scheme, composed of 15 traditional varieties of cowpea and two salinity levels of irrigation water (0.5 and 4.5 dS m^−1^), with five replicates. Plants were grown in pots containing 10 dm^3^ of soil for 80 days. The reduction in the photosynthetic rate of cowpea varieties occurs mainly due to the decrease in stomatal conductance caused by salt stress. Salt stress increased the electron transport rate and photochemical quenching of cowpea varieties, but stress-tolerant varieties increased the CO_2_ assimilation rate and instantaneous carboxylation efficiency. The Ceará, Costela de Vaca, Pingo de Ouro, Ovo de Peru, and Sempre Verde varieties are tolerant to salt stress. Salt stress decreases 26% of the production of tolerant varieties to salt stress and 54% of susceptible varieties. The present findings show the existence of variability for saline stress tolerance in traditional varieties of cowpea and that Ceará, Costela de Vaca, Pingo de Ouro, and Ovo de Peru varieties are more suitable for crops irrigated with saline water.

## 1. Introduction

Cowpea beans (*Vigna unguiculata* (L.) Walp.), also known as rope or Macassar beans, are a significant source of protein in the north and northeastern regions of Brazil [1]. Family and small farms are the leading producers, using mostly native or traditional seeds [2]. These farmers have little technological apparatus and use family labor and seeds from selection made by the farmer himself, with well-defined and recognized phenotypic characteristics that characterize them as native or traditional seeds. Traditional or local varieties are highly adapted to the places where they are conserved and managed, and they are part of family autonomy, constituting the main factor in people’s food security.

Farms in the northeastern region, especially those in Mossoró/Açu, RN, Brazil, require a substantial amount of water, which has driven the use of saline water up to 4.5 dS m^−1^, e.g., groundwater from the Calcário Jandaíra aquifer [3]. Cowpea beans are moderately tolerant to salinity, up to 3.3 dS m^−1^ in irrigation water and 4.8 dS m^−1^ in the soil [4]. However, it is necessary to use saline water due to the scarcity of good quality water and the increase in water consumption to meet population growth and irrigated agriculture.

Water with a high sodium adsorption ratio (SAR) can modify the physicochemical conditions of the soil, and excess soluble salts result in lower osmotic potential, water deficit, stomatal closure, limited CO_2_ assimilation/water usage, and alterations to the photochemical process [5,6]. Osmotic restrictions combined with ionic restrictions and nutritional imbalance limit gas exchange and biomass accumulation and production [5,6,7,8]. The decrease in productivity of cowpea under saline stress occurs due to the decrease in the water potential, survival rate, plant’s initial vigor, growth, and photosynthetic activity and excessive accumulation of Cl and Na ions and reactive oxygen species (ROS) [5,6,7,8,9,10,11]. Therefore, studies that evaluate gas exchange and photosynthetic efficiency are important for the identification of salinity-tolerant plants.

Identifying and selecting salinity-tolerant cowpea bean varieties can facilitate the use of saline water without affecting cowpea production. While there are early growth stage studies that designate salinity-tolerant cowpea bean genotypes [2,12,13], studies investigating the complete production cycle are lacking. In this study, we hypothesized that the genetic diversity of cowpea beans grown in the semi-arid region would create salinity-tolerant cowpea bean varieties that may be cultivated in farms with poor water quality. Thus, the goals of this study were to assess the effect of irrigation with brackish water on the growth, gas exchange, and production of 15 traditional varieties of cowpea and determine the most saltwater-tolerant varieties.

## 2. Results

### 2.1. Soil Salinity

There was a significant interaction between varieties and irrigation-water salinity levels (*p* < 0.05) (Table 1). Irrigation with saline water of 4.5 dS m^−1^ increased the ECse of the soil contained in the pots cultivated with the plants of cowpea varieties compared to pots that received the water of 0.5 dS m^−1^ (control). The ECse of the soil contained in the pots irrigated with high-salinity water varied between 6.3 and 9.7 dS m^−1^, that is, between 1.4 and 2.15 times the salinity of the irrigation water. When irrigation was performed with high-salinity water in pots cultivated with the varieties Canário and Roxão, the highest values of ECse in the soil were recorded, 9.7 and 8.4 dS m^−1^, respectively, with no difference for salinity in pots cultivated with the other varieties (Table 1).

### 2.2. Gas Exchange

The interaction between varieties and salinity levels was significant for the net CO_2_ assimilation rate (*p* < 0.01), internal CO_2_ concentration (*p* < 0.01), and instantaneous carboxylation efficiency (*p* < 0.01) (Table 2).

The *A_N_* of the varieties Boquinha, Roxão, Feijão Branco, Canapu Miúdo, Coruja, and Paulistinha was reduced under salt stress (4.5 dS m^−1^), varying, on average, between 13.19 and 74.21% compared to the control (0.5 dS m^−1^). The varieties Ceará, Canário, Pingo de Ouro, Canapu Branco, Baeta, and Sempre Verde had increased *A_N_* in the saline treatment (4.5 dS m^−1^) with an average variation between 11.95 and 56.71% compared to the control (0.5 dS m^−1^). Salt stress did not influence (*p* < 0.05) the *A_N_* of the varieties Costela de Vaca, Lisão, and Ovo de Peru (Table 2).

There was the formation of three clusters regarding photosynthetic performance under the salt-stress condition, with the varieties Sempre Verde, Costela de Vaca, Pingo de Ouro, and Baeta in the cluster of higher *A_N_*, Ceará, Lisão, Canário, Canapu Branco, Boquinha, Roxão, and Paulistinha in the cluster of intermediate *A_N_*, and Ovo de Peru, Feijão Branco, Coruja, and Canapu Miúdo in the cluster of low *A_N_*, in this sequence (Table 2).

The application of 4.5 dS m^−1^ water reduced the *Ci* in the varieties Canapu Branco, Ovo de Peru, and Baeta compared to the control (0.5 dS m^−1^), with reductions ranging between 11.80 and 23.05%. A different behavior was observed in the varieties Ceará, Canário, Pingo de Ouro, Canapu Miúdo, Coruja, and Sempre Verde, which had an average increment ranging between 11.82 and 42.68% in *Ci* under salt stress (4.5 dS m^−1^) compared to the control (0.5 dS m^−1^). The varieties Boquinha, Costela de Vaca, Lisão, Roxão, Feijão Branco, and Paulistinha did not have their *Ci* influenced by salt stress (Table 2).

The *CEi* of the varieties Pingo de Ouro, Canapu Branco, Baeta, and Sempre Verde were increased under the salt-stress conditions compared to the control, with a variation between 33.93 and 64.15%. The varieties Boquinha, Feijão Branco, Canapu Miúdo, Coruja, and Paulistinha had reduced *CEi*, between 19.64 and 83.08%, on average, under the salt-stress conditions compared to the control (Table 2). The varieties Ceará, Costela de Vaca, Lisão, Canário, Roxão, and Ovo de Peru did not have their *CEi* influenced by salt stress (Table 2). The best *CEi* values under the salt-stress conditions were observed in the varieties Costela de Vaca, Pingo de Ouro, Baeta, and Sempre Verde. The worst *CEi* values under the salt-stress conditions were observed in the varieties Feijão Branco, Canapu Miúdo, Ovo de Peru, and Coruja (Table 2).

The interaction between varieties and salinity levels was significant for stomatal conductance (*p* < 0.01), transpiration (*p* < 0.01), and instantaneous water use efficiency (*p* < 0.01) (Table 3).

When comparing the conditions of salt stress (4.5 dS m^−1^) and control (0.5 dS m^−1^) for *gs* (Table 3), the Boquinha, Roxão, Feijão Branco, Canapu Branco, Canapu Miúdo, Ovo de Peru, Coruja, and Paulistinha varieties had an average reduction ranging between 7.69 and 70.00%. The Ceará, Canário, Pingo de Ouro, and Sempre Verde varieties showed an average increase ranging between 62.50 and 100.00%. On the other hand, the stomatal conductance values of the Costela de Vaca, Lisão, and Baeta varieties were not influenced by salt stress. Under this condition, the lowest values of *gs* were verified in the Canapu Miúdo, Ovo de Peru, Roxão, Feijão Branco, and Coruja varieties, while the highest values were recorded in the varieties Sempre Verde and Ceará.

Except for the Ceará, Canário, and Sempre Verde varieties, which had an increase in *E*, the others showed a reduction in *E*, ranging from 15.68 to 65.64% on average, under the condition of salt stress (4.5 dS m^−1^) compared to the control (0.5 dS m^−1^). Under the salt-stress condition, the lowest *E* values were verified in the Canapu Miúdo, Baeta, Canapu Branco, and Roxão varieties (Table 3).

The *WUEi* of the Canapu Miúdo and Coruja varieties decreased on average by 23.69% under the condition of salt stress (4.5 dS m^−1^) compared to the control (0.5 dS m^−1^) (Table 3). Under the condition of salt stress, there was an average increase between 18.79 and 187.80% in the *WUEi* of the Boquinha, Costela de Vaca, Lisão, Pingo de Ouro, Roxão, Feijão Branco, Canapu Branco, Ovo de Peru, Baeta, and Sempre Verde varieties compared to the control treatment. The Ceará, Canário, and Paulistinha varieties did not have their *WUEi* influenced by salt stress (Table 3). Under this condition, the highest values of *WUEi* were verified in the Baeta, Costela de Vaca, Pingo de Ouro, and Canapu Branco varieties, while the lowest values were found in the Canapu Miúdo, Coruja, Ceará, Feijão Branco, and Paulistinha varieties (Table 3).

### 2.3. Chlorophyll Fluorescence

There were significant effects of water salinity levels on initial fluorescence (*p* < 0.05), variable fluorescence (*Fv*) (*p* < 0.05), the maximum quantum efficiency of photosystem II (*p* < 0.01), the quantum efficiency of photosystem II (*p* < 0.01), electron transport rate (*p* < 0.01), and quantum yield of regulated photochemical quenching (*p* < 0.01) (Table 4).

The cowpea varieties irrigated with high-salinity water (4.5 dS m^−1^) showed increments in the values of *Fv*, *Fv/Fm*, *ETR,* and *Y (NPQ)* of 3.29, 1.33, 79.02, and 11.11% compared to the control treatment (0.5 dS m^−1^), respectively (Table 4). However, irrigation with high-salinity water (4.5 dS m^−1^) reduced *Fo* and *Y(II)* by 3.65 and 4.41% compared to the control treatment (0.5 dS m^−1^), respectively (Table 4).

### 2.4. Growth and Biomass Accumulation

There were simple effects of salinity levels and varieties for stem diameter and number of leaves (Table 5). Irrigation with high-salinity water decreased on average by 14.44 and 50.0% the stem diameter (SD) and the number of leaves (NL), respectively, of cowpea plants compared to the control (Table 5). The varieties Boquinha, Ceará, Canapu Miúdo, and Ovo de Peru had the highest SD, while Ceará showed the highest NL regardless of the water salinity level (Table 5).

**Figure 1:**
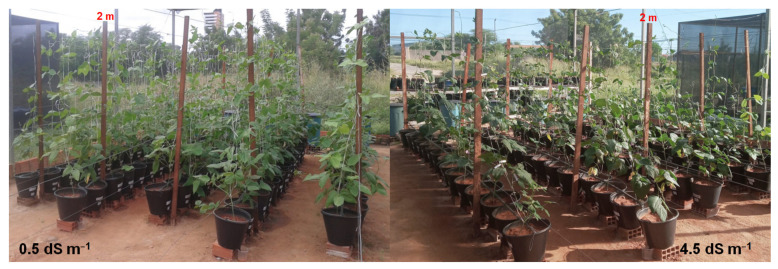
Traditional varieties of cowpea subjected to two levels of irrigation-water salinity.

There was a significant interaction between varieties and salinity levels for main branch length (*p* < 0.01) and shoot dry mass (*p* < 0.05) (Table 6). Main branch length (*MBL*) was reduced, with an average variation between 26.52 and 63.73% in the varieties Costela de Vaca, Pingo de Ouro, Roxão, Canapu Branco, Canapu Miúdo, and Baeta, when irrigated with water of 4.5 dS m^−1^ compared to the control (Table 6, Figure 1). Among the varieties irrigated with saline water, Ceará, Canário, Roxão, and Ovo de Peru had the highest *MBL* values (Table 6).

Irrigation with water of 4.5 dS m^−1^ reduced shoot dry mass (*SDM*) in all cowpea varieties, between 24.16 and 58.36%, compared to those irrigated with water of 0.5 dS m^−1^ (Table 6, Figure 1). Under the condition of irrigation with water of 4.5 dS m^−1^, the highest accumulations of shoot dry mass (Table 6) were recorded in the varieties Boquinha, Canário, Pingo de Ouro, Roxão, Feijão Branco, Ovo de Peru, and Baeta.

### 2.5. Grain Production

The interaction between salinity levels and cowpea varieties was significant (*p* < 0.01) for the number of pods per plant (NPP), the number of seeds per pod (NSPo), the number of seeds per plant (NSPl), and production per plant (PP) (Table 7).

The number of pods per plant (NPP), the number of seeds per plant (NSPl), and production per plant (PP) were reduced by up to 68.89, 71.96, and 61.26% on average, respectively, under irrigation with water of 4.5 dS m^−1^ compared to water of 0.5 dS m^−1^, in all cowpea varieties except for Ceará, Costela de Vaca, Pingo de Ouro, Ovo de Peru, and Sempre Verde, whose production per plant (PP) was not influenced by salinity (Table 7). However, there was no difference in PP between cowpea varieties when irrigated with high-salinity water (Table 7).

The number of seeds per pod (NSPo) of the varieties Canário, Roxão, and Coruja was reduced by up to 30.07% by irrigation with saline water, but the NSPo of the Boquinha variety was increased by 31.90% under salt stress compared to the control (Table 7).

### 2.6. Salinity Tolerance

**Figure 2:**
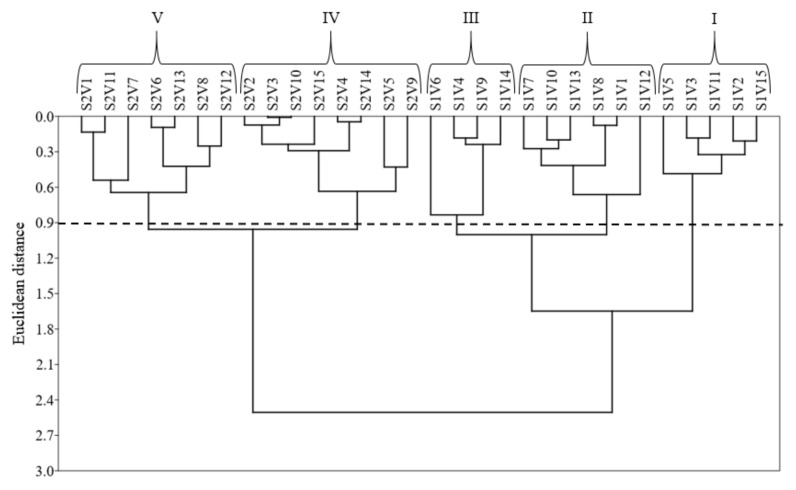
Dissimilarity dendrogram of the clusters formed by the combination of salinity levels (S) and traditional varieties of cowpea (V). S1—low salinity (0.5 dS m^−1^). S2—high salinity (4.5 dS m^−1^). V1—Boquinha, V2—Ceará, V3—Costela de Vaca, V4—Lisão, V5—Canário, V6—Pingo de Ouro, V7—Roxão, V8—Feijão Branco, V9—Canapu Branco, V10—Canapu Miúdo, V11—Ovo de Peru, V12—Baeta, V13—Coruja, V14—Paulistinha, and V15—Sempre Verde.

In the cluster analysis, based on the Euclidean distance of 0.90 in the formation of five clusters of combinations between salinity levels (S) and cowpea varieties (V) (Figure 2), the first three clusters (I) are characterized by the 15 varieties of cowpea irrigated with low-salinity water (0.5 dS m^−1^). Those irrigated with high-salinity water (4.5 dS m^−1^) were grouped in clusters IV and V. Cluster two (IV) comprises the varieties V2 (Ceará), V3 (Costela de Vaca), V4 (Lisão), V5 (Canário), V9 (Canapu Branco), V10 (Canapu Miúdo), V14 (Paulistinha), and V15 (Sempre Verde). The third cluster (V) contains the varieties V1 (Boquinha), V6 (Pingo de Ouro), V7 (Roxão), V8 (Feijão Branco), V11 (Ovo de Peru), V12 (Baeta), and V13 (Coruja) (Figure 2).

## 3. Discussion

The soil salinity limit for cowpea culture is 4.8 dS m^−1^ [4]. Irrigation water with 4.5 dS m^−1^ increased by 1.4 to 2.15 times the electrical conductivity of the soil saturation extract considering the soil salinity limit. Therefore, every cowpea variety was under possible salt stress. This behavior was similar to that observed in irrigated areas with lower leaching fractions where irrigation-water salinity influenced the soil salinity at the end of the cycle [7,14].

Soil from pots with the highest SDM levels presented the highest ECse values (Canário and Roxão). This behavior relates to the diversity of salinity tolerance mechanisms [15], e.g., water consumption restrictions, selective salt absorption, and salt exclusion at the roots. Soil from pots with the lowest SDM levels exhibited the lowest ECse values (Costela de Vaca, Lisão, Canapu Miúdo, and Paulistinha) because of vacuolar ion compartmentation and non-selective ion absorption in the most susceptible varieties [16,17].

Irrigation water with 4.5 dS m^−1^ reduced the photosynthetic rate (*A_N_*) in the Boquinha, Roxão, Feijão Branco, Canapu Miúdo, Coruja, and Paulistinha varieties. The *A_N_* reductions were related to stomatal factors, i.e., the reduction of stomatal conductance limited the influx of CO_2_ and consequently the internal concentration of CO_2_ (*Ci*) in the substomatal cavity, reducing water absorption and transpiration [6,17,18]. The reduction of stomatal conductance occurred with increased soil salt concentrations and led to a decrease in osmotic and water potentials, resulting in toxicity of specific ions—Na^+^ and Cl^-^ [5,8,9]. Plants experience issues absorbing water from the soil under salt stress and tend to reduce water loss by closing stomates and reducing transpiration [6,18].

The reduced A_N_ in the Boquinha, Feijão Branco, Canapu Miúdo, Coruja, and Paulistinha varieties was also related to non-stomatal factors. There were reductions in the *A/Ci*, indicating decreased ribulose 1,5-bisphosphate carboxylase/oxygenase (Rubisco) enzymic activity under stress conditions, e.g., the lack of adenosine triphosphate (ATP) and nicotinamide adenine dinucleotide phosphate (NADPH) from the electron transport chain of photosystem II [6,19].

The Ceará, Canário, Pingo de Ouro, Canapu Branco, Baeta, and Sempre Verde varieties increased A_N_ under salt stress. The increased production of photo-assimilates under salt stress improves the energetic input and allows the plant to use mechanisms to tolerate energy expenditure, such as vacuolar ion compartmentation, the exclusion of specific ions, and attempting to attain ionic homeostasis [16,17].

The increased *A_N_* in the Ceará, Canário, Pingo de Ouro, Canapu Branco, Baeta, and Sempre Verde varieties coincided with increases in *gs*, CO_2_ influx, transpiration, and the consequent water absorption. Increased water loss because of transpiration results in reduced water potential at the roots, overcoming the osmotic stress and aiding in water absorption [9,10,11]. The *A_N_* of the Pingo de Ouro, Canapu Branco, Baeta, and Sempre Verde varieties coincided with increased *A_N_/Ci* and *WUEi*, improved water usage, and increased Rubisco activity. However, the increased *A_N_* in the Canapu Branco variety also coincided with a decrease in gs and E; in this case, the higher salt concentration, improved water usage (*WUEi*), and increased Rubisco activity (*A/Ci*) resulted in water consumption restrictions.

The *A_N_* of the Costela de Vaca, Lisão, and Ovo de Peru varieties was not affected by salinity, but the *WUEi* increased because of lower transpiration. Soil salinity impairs water absorption by cowpea bean plants; therefore, lower transpiration is a strategy to reduce water losses [6,18].

Chlorophyll fluorescence did not differ among varieties, indicating similar photochemical activities. Increases of *Fv* and *ETR* in cowpea bean varieties under salt stress compared with control demonstrated an increased ability to transfer energy from the excited electrons of chlorophyll molecules to assemble NADPH and ATP and had reduced ferredoxin (Fdr). This increased energy transfer was vital for preventing the decrease in photosynthesis or improving photosynthesis under salt stress once the quantum efficiency of photosystem II (*Y(II)*) in cowpea bean varieties decreased under salt stress, indicating a decrease in the fraction of energy absorption by chlorophyll in PSII [20]. However, some varieties susceptible to salt stress, e.g., Boquinha, Roxão, Feijão Branco, Canapu Miúdo, Coruja, and Paulistinha, exhibited decreased *A_N_* and *A/Ci*, despite this mechanism for increasing the photochemical energy transfer.

Cowpea bean plants increased their photoprotective capability under salt stress because of a higher quantum yield of regulated photochemical quenching (*Y_(NPQ)_*) through thermal energy dissipation by the xanthophyll cycle [21]. This photoprotective mechanism is efficient in cowpea bean plants once the *Fv/Fm* values are greater than 0.75, indicating a lack of degradation of the photosynthetic apparatus [17]. Compared with the control, the *Fo* values were lower under salt stress, corroborating the absence of damage in the PSII reaction centers [20]. 

Despite improvements in *A_N_*, *WUE*, and *A/Ci,* the growth, biomass accumulation, and production of cowpea beans decreased under salt stress, especially the primary branch length in the Costela de Vaca, Pingo de Ouro, Roxão, Canapu Branco, Canapu Miúdo, and Baeta varieties, characterized by their prostrate and semi-prostrate size and indeterminate growth habit. The reduced growth resulted from lower energetic stability because of decreased *Y_(II)_* under salt stress. The authors in [22] reported that reduced biomass in cowpea bean plants under salt stress relates to an energy bypass because of the metabolic cost incurred during acclimation.

In plants, salt stress results in morphologic and anatomic modifications with strategies for adapting to adverse conditions, e.g., fewer leaves and shorter branches, reflecting decreased transpiration to improve water absorption [14,23].

Roxão was the only variety without SDM modifications caused by salinity and was unique in the susceptible group with an investment in biomass at the expense of grain production. The authors in [7,8,24,25] and other authors observed reduced growth and biomass accumulation in cowpea beans under salt stress. These reductions are part of the acclimation process of cowpea bean plants that have the potential to tolerate salt stress as they seek to secure production and perpetuate the species. In this sense, varieties such as Ovo de Peru, Pingo de Ouro, Sempre Verde, Costela de Vaca, and Ceará presented reduced growth and biomass accumulation but exhibited the highest production compared with the control.

Grain production was reduced (*p* < 0.05) in cowpea bean varieties under salt stress, except for the Ceará, Costela de Vaca, Pingo de Ouro, Ovo de Peru, and Sempre Verde varieties. Reduced water absorption associated with specific ion toxicity and physiological effects of salinity resulted in reduced growth and production [6,26]. The stress from salt accumulation in plants resulted in fewer reproductive branches and higher abortion rates [14,27]. All varieties produced fewer pods under salt stress; however, the Ceará, Pingo de Ouro, and Sempre Verde had more seeds per pod, compensating for the final grain production.

Cluster analysis revealed heterogenicity among plants under saline water irrigation with the Ceará, Costela de Vaca, Lisão, Canário, Canapu Branco, Canapu Miúdo, Paulistinha, and Sempre Verde varieties presenting similar SDM and production compared with the control, indicating tolerance to high irrigation-water salinity. The tolerance of Lisão, Canário, Canapu Branco, Canapu Miúdo, and Paulistinha occurred due to SDM. The Ceará, Costela de Vaca, and Semper Verde varieties are more tolerant to saline stress for SDM and grain production. We recommend the Ceará, Costa de Vaca, Pingo de Ouro, Ovo de Peru, and Semper Verde varieties for grain production under saline stress conditions. Those results differed from studies with conventional cowpea bean varieties under salt stress, e.g., [28] (EPACE 10); [29] (MNC04-762F-9, MNC04-762F-3, MNC04-762F-21, MNC04-769F-62, and MNC04-765F-153); [30] (IPA-206 and BRS Guariba); [27] (BRS Pajeu); [24] (BRS Imponente, MNC04-795F-168, and MNC04-795F-161); [31] (CE 790 and CE 104); [32] (BRS Pajeú); and [33] (BRS Itaim), who reported that these conventional varieties were susceptible to high irrigation-water salinity (5.0, 4.8, 5.0, 4.5, 6.4, 5.0, 6.0, 4.5, and 6.0 dS m^−1^, respectively). These findings support the hypothesis that traditional varieties are more tolerant of salt stress than conventional varieties; however, further field studies are necessary. The Boquinha, Pingo de Ouro, Roxão, Feijão Branco, Ovo de Peru, Baeta, and Coruja varieties presented significantly different values compared to the control, indicating high susceptibility to irrigation-water salinity (4.5 dS m^−1^).

In summary, the reduced photosynthetic rates in cowpea bean varieties are mainly caused by reductions in stomatal conductance resulting from salt stress. Salt stress increases the energy transferability of photosystem II in cowpea bean varieties, increasing the CO_2_ assimilation rate and the instantaneous carboxylation efficiency in varieties more tolerant to salt stress. Salt stress decreases 26% of the production of tolerant varieties to salt stress and 54% of susceptible varieties. The Ceará, Costela de Vaca, Pingo de Ouro, Ovo de Peru, and Sempre Verde varieties exhibited the best physiological and production performance under salt stress; therefore, these varieties are tolerant to salt stress. The Lisão, Canário, Canapu Branco, Canapu Miúdo, Paulistinha, Boquinha, Roxão, Feijão Branco, Baeta, and Coruja present the worst physiological and production performances under salt stress; therefore, those varieties are susceptible to salt stress.

## 4. Material and Methods

### 4.1. Location, Experimental Design, and Plant Material

The experiment was conducted in a greenhouse at the Federal Rural University of the Semi-Arid Region—UFERSA, East campus, Mossoró/RN, Brazil, from May to August 2019. The municipality is located at the geographical coordinates of 5°12′ S and 37°19′ W, with an average altitude of 18 m. According to Köppen’s classification, the climate of the region is BSwh’, and maximum and minimum temperatures of 44.2 and 20.4 °C and maximum and minimum relative humidity (RH) of 86 and 22%, respectively, were recorded during the experimental period. The average temperature and average daily relative humidity throughout the experiment were 33.8 °C and 49% RH, respectively.

The experimental design used was randomized blocks, with treatments arranged in a 15 × 2 factorial scheme, consisting of the combination of fifteen cowpea varieties (V1 (Boquinha), V2 (Ceará), V3 (Costela de Vaca), V4 (Lisão), V5 (Canário), V6 (Pingo de Ouro), V7 (Roxão), V8 (Feijão Branco), V9 (Canapu Branco), V10 (Canapu Miúdo), V11 (Ovo de Peru), V12 (Baeta), V13 (Coruja), V14 (Paulistinha), and V15 (Sempre Verde)) with two levels of salinity of irrigation water (0.5 dS m^−1^ and 4.5 dS m^−1^), with five replicates.

The seeds used were acquired from collections from Traditional Seed Guardians belonging to rural communities located in municipalities of the western region of the Rio Grande do Norte state. The seeds came from the 2018 season and were stored in PET bottles, which were sealed to avoid any change in the degree of moisture and stored in dry, well-ventilated warehouses without the use of preservatives. The varieties used in this study were chosen based on a preliminary study conducted on the germination and initial growth stages of cowpea [2].

### 4.2. Experiment Setup and Fertilization Management

Sowing was performed using 9 seeds, with the first thinning performed at 4 days after germination, leaving 3 plants per pot, and the second thinning 15 days later, leaving only one plant per pot.

Each experimental unit consisted of a plastic pot with a capacity of 12.0 L, with 1.0 L filled with crushed stone at the bottom, 1.0 L free at the top, and 10.0 L filled with soil classified as *Latossolo Vermelho Amarelo distrófico* (Oxisol), sandy loam texture [34], whose physical and chemical characteristics are presented in Table 8.

Soil acidity was corrected with calcium hydroxide (Ca(OH)_2_), with 54% calcium. The soil was corrected to increase base saturation to 90%. After 15 days, the soil was fertilized according to the recommendations of [35] for pots in protected cultivation, applying 300 mg of P_2_O_5_^−^, 150 mg of K_2_O, and 100 mg of N per dm^3^ of soil through fertigation, using urea (45% of N), potassium chloride (KCl = 60% of K_2_O), and monoammonium phosphate (MAP = 12% of N and 50% of P_2_O_5_^−^). Fertilization with micronutrients was performed by foliar application in pre-flowering and 15 days after flowering, with the foliar fertilizer Liqui-Plex Fruit^®^ in the proportion of the 3 mL L^−1^ of the solution, following the manufacturer’s recommendation (Table 9).

### 4.3. Saline Waters and Irrigation and Drainage Management

In the preparation of irrigation waters, local-supply water (ECw = 0.50 dS m^−1^) was used for the lowest level of salinity. For the highest level of salinity (ECw = 4.50 dS m^−1^), local-supply water was mixed with reject brine from brackish water desalination (ECw = 9.50 dS m^−1^). The desalination reject brine was obtained at the Jurema Settlement, located beside the RN-013 highway, km 4 (Table 10). Local supply water and brine tailings were stored in water tanks with a volume of 2000 L. We monitored the electrical conductivity during mixing with a portable conductivity meter.

Irrigation management was based on the drainage lysimeter method to leave the soil with moisture close to the maximum retention capacity, and irrigations were performed once a day, applying a leaching fraction (LF) of 15% every seven days along with the applied depth. The volume applied (Va) per container was obtained by the difference between the previous depth applied (La) minus the mean drainage (D), divided by the number of containers (n), as indicated in Equation (1):

(1) $$Va=\frac{La-D}{n(1-LF)}$$

The irrigation system comprised a self-venting Metalcorte/Eberle circulation motor pump, driven by a single-phase motor, 210 V voltage, 60 Hz frequency, installed in a reservoir with a capacity of 50 L and 16 mm diameter hoses with pressure-compensating drippers with a flow rate of 1.3 L h^−1^.

The electrical conductivity of the saturation extract (ECse) was estimated according to the methodology suggested by [4] for medium-textured soils. For this, at 80 days after sowing, an additional leaching fraction (15%) was applied, the drained volume was collected, and the electrical conductivity of the drainage water (ECd) was measured using a benchtop conductivity meter, with the data expressed in dS m^−1^ adjusted to the temperature of 25 °C. The data were applied in Equation (2):

(2) $$ECse=\frac{ECd}{2}$$

### 4.4. Analysis of Gas Exchange and Chlorophyll a Fluorescence

Physiological analyses were performed during the flowering stage of the plants, at 58 days after sowing. Gas exchange was analyzed in the period from 6 to 9 a.m., with evaluations on fully expanded leaves located in the upper third of each plant, using a portable infrared gas analyzer (IRGA), LCPro^+^ Portable Photosynthesis System^®^ (ADC BioScientific Limited, Hertfordshire, UK) with temperature control at 25 °C, irradiation of 1200 μmol photons m^−2^ s^−1^, and airflow of 200 mL min^−1^. The quantified variables were CO_2_ assimilation rate (*A_N_*) (µmol (CO_2_) m^−2^ s^−1^), transpiration (*E*) (mmol (H_2_O) m^−2^ s^−1^), stomatal conductance (*gs*) (mol (H_2_O) m^−2^ s^−1^), and internal CO_2_ concentration (*Ci*) (mol m^−2^ s^−1^). These data were then used to estimate the instantaneous water use efficiency (*WUEi*) (*A_N_*/*E*) [(µmol (CO_2_) m^−2^ s^−1^) (mmol (H_2_O) m^−2^ s^−1^)^−1^] and instantaneous carboxylation efficiency (*CEi*) (*A_N_/Ci*) [(µmol (CO_2_) m^−2^ s^−1^) (mol (CO_2_) m^−2^ s^−1^)^−1^] [6].

Immediately after gas exchange measurements, chlorophyll *a* fluorescence was evaluated using the OS5p pulse-modulated fluorometer from Opti science; the Fv/Fm protocol was used for evaluations under dark conditions. Under these conditions, the following fluorescence induction variables were estimated: initial fluorescence (*Fo*) (µmol (photons) m^−2^ s^−1^), maximum fluorescence (*Fm*) (µmol (photons) m^−2^ s^−1^), variable fluorescence (*Fv = Fm-Fo*) (µmol (photons) m^−2^ s^−1^), and the maximum quantum efficiency of PSII (*Fv/Fm*) [6].

The pulse-modulated fluorometer was also used to perform evaluations under light conditions, through the yield protocol. Readings were taken by applying the actinic light source with a multi-flash saturating pulse, coupled to a photosynthetically active radiation determination clip (PAR-Clip) to estimate the following variables: initial fluorescence before the saturation pulse (*F’*), maximum fluorescence after adaptation to saturating light (*Fm’*), electron transport rate (*ETR*) (µmol (photons) m^−2^ s^−1^), and quantum efficiency of photosystem II (*Y(II)*). With these data, the following parameters were determined: minimum fluorescence of the illuminated plant tissue (Fo’) [36], photochemical quenching coefficient by the lake model (*qL*) [37], quantum yield of regulated photochemical quenching (*Y(NPQ)*) [37], and the quantum yield of non-regulated photochemical quenching (*Y(NO)*) [37].

### 4.5. Growth Analysis and Biomass Accumulation

At 58 DAP, the following parameters were determined: main branch length (MBL), using a measuring tape and measured from the plant collar to the last leaf insertion; stem diameter (SD), measured at 1.0 cm from the plant collar using a digital caliper; and the number of leaves (NL). After harvesting the pods of all varieties 80 days after sowing, the aerial part of the plants was collected and dried in an oven with forced air circulation, at a temperature of 65 °C, until reaching constant weight, to quantify the values of shoot dry mass (SDM).

### 4.6. Production Quantification

The pods were harvested as each traditional variety reached the phenological stage R9 (maturity stage), when the fruits were dry with the color and brightness that are characteristic of the genotype. The pods were transported to the laboratory, where the number of pods per plant (NPP), the number of seeds per pod (NSPo), the number of seeds per plant (NSPl), and production per plant (PP) (g) were counted.

### 4.7. Statistical Analysis

The data were subjected to analysis of variance and F-test. In cases of significant effect, the Scott–Knott test (*p* < 0.05) was performed for the variety factor, and Student’s *t*-test (*p* < 0.05) was performed for the salinity factor, using SISVAR^®^ statistical analysis software [38]. The data of shoot dry mass and grain production per plant were used to classify salinity tolerance; for this, the data were subjected to standardization, leaving mean zero ($\bar{X}$ = 0) and variance one (S^2^ = 1). Subsequently, cluster analysis was performed by hierarchical method, Ward’s minimum variance, using the Euclidean distance as a measure of dissimilarity. PAST 3 free software was used for univariate and multivariate statistical analyses.

## Figures and Tables

**Table 1:** F-test and means test for electrical conductivity of saturation extract (ECse, dS m^−1^) of soils cultivated with traditional varieties of cowpea subjected to two levels of irrigation-water salinity.

F-Test (*p*-Value)		
Sources of Variation	ECse	
Block	0.061	
Salinity	0.000	
Varieties	0.011	
Salinity × Varieties	0.018	
Means comparison test (Standard Deviation, *n* = 5)		
Varieties	ECse	
	0.5 dS m^−1^	4.5 dS m^−1^
V1—Boquinha	1.3 ± 0.19 Ba	7.8 ± 0.35 Ac
V2—Ceará	1.1 ± 0.11 Ba	7.3 ± 0.72 Ac
V3—Costela de Vaca	1.4 ± 0.23 Ba	6.7 ± 0.42 Ac
V4—Lisão	1.5 ± 0.20 Ba	6.5 ± 0.69 Ac
V5—Canário	1.4 ± 0.20 Ba	9.7 ± 0.79 Aa
V6—Pingo de Ouro	1.4 ± 0.22 Ba	7.8 ± 0.40 Ac
V7—Roxão	1.7 ± 0.17 Ba	8.4 ± 0.34Ab
V8—Feijão Branco	1.4 ± 0.08 Ba	7.8 ± 0.41 Ac
V9—Canapu Branco	1.5 ± 0.17 Ba	7.4 ± 0.48 Ac
V10—Canapu Miúdo	1.3 ± 0.17 Ba	6.7 ± 0.64 Ac
V11—Ovo de Peru	1.3 ± 0.21 Ba	7.9 ± 1.07 Ac
V12—Baeta	1.9 ± 0.13 Ba	7.2 ± 0.41 Ac
V13—Coruja	1.3 ± 0.05 Ba	7.0 ± 0.73 Ac
V14—Paulistinha	1.4 ± 0.10 Ba	6.3 ± 0.47 Ac
V15—Sempre Verde	1.4 ± 0.19 Ba	7.5 ± 0.38 Ac

Equal uppercase letters in the rows and lowercase letters in the column do not differ by Student’s *t*-test and Scott–Knott test at 5% probability level, respectively.

**Table 2:** F-test and means test for net CO_2_ assimilation rate (*A_N_*, µmol (CO_2_) m^−2^ s^−1^), internal CO_2_ concentration (*Ci*, mol (CO_2_) m^−2^ s^−1^), and instantaneous carboxylation efficiency (*CEi*, (µmol (CO_2_) m^−2^ s^−1^) (mol (CO_2_) m^−2^ s^−1^)^−1^) of traditional varieties of cowpea subjected to salinity levels of irrigation water.

F-Test (*p*-Value)						
Sources of Variation	*A_N_*		*Ci*		*CEi*	
Block	0.543		0.000		0.0284	
Salinity	0.483		0.000		0.006	
Varieties	0.000		0.000		0.000	
Salinity × Varieties	0.000		0.000		0.000	
Means comparison test (Standard Deviation, *n* = 5)						
Varieties	*A_N_*		*Ci*		*CEi*	
	0.5 dS m^−1^	4.5 dS m^−1^	0.5 dS m^−1^	4.5 dS m^−1^	0.5 dS m^−1^	4.5 dS m^−1^
V1—Boquinha	16.92 ± 0.37 Aa	10.13 ± 0.82 Be	187 ± 6.67 Ac	198 ± 13.50 Ab	0.091 ± 0.003 Aa	0.053 ± 0.007 Bd
V2—Ceará	8.15 ± 0.08 Be	11.56 ± 0.90 Ad	198 ± 3.27 Bc	239 ± 8.21 Aa	0.041 ± 0,001 Ad	0.049 ± 0.005 Ad
V3—Costela de Vaca	15.04 ± 0.34 Ab	15.11 ± 0.38 Ab	155 ± 5.76 Ad	164 ± 3.67 Ad	0.098 ± 0.006 Aa	0.092 ± 0.004 Aa
V4—Lisão	11.77 ± 0.03 Ac	12.27 ± 0.49 Ad	195 ± 2.03 Ac	200 ± 4,52 Ab	0.060 ± 0.001 Ac	0.062 ± 0.004 Ac
V5—Canário	7.39 ± 0.54 Be	11.12 ± 0.16 Ad	137 ± 11.98 Be	174 ± 4.06 Ad	0.057 ± 0.009 Ac	0.064 ± 0.002 Ac
V6—Pingo de Ouro	8.34 ± 0.61 Be	13.07 ± 0.27 Ac	165 ± 6.27 Bd	189 ± 4.23 Ac	0.051 ± 0.005 Bc	0.069 ± 0.003 Ab
V7—Roxão	11.58 ± 0.12 Ac	9.81 ± 0.82 Be	190 ± 6.16 Ac	192 ± 8.08 Ac	0.061 ± 0.002 Ac	0.052 ± 0.007 Ad
V8—Feijão Branco	10.78 ± 0.12 Ad	7.98 ± 0.06 Bf	215 ± 3.85 Ab	208 ± 7.00 Ab	0.050 ± 0.001 Ac	0.039 ± 0.001 Be
V9—Canapu Branco	9.96 ± 0.42 Bd	11.15 ± 0.30 Ad	243 ± 2.94 Aa	187 ± 7.19 Bc	0.041 ± 0.002 Bd	0.060 ± 0.004 Ac
V10—Canapu Miúdo	10.47 ± 0.40 Ad	2.70 ± 0.19 Bg	164 ± 6.03 Bd	234 ± 10.72 Aa	0.065 ± 0.005 Ac	0.011 ± 0.001 Bf
V11—Ovo de Peru	8.35 ± 0.14 Ae	8.03 ± 0.27 Af	231 ± 1.88 Aa	203 ± 3.25 Bb	0.036 ± 0.001 Ad	0.040 ± 0.002 Ae
V12—Baeta	9.27 ± 0.24 Be	13.61 ± 0.16 Ac	178 ± 9.71 Ad	157 ± 5.34 Bd	0.053 ± 0.002 Bc	0.087 ± 0.003 Aa
V13—Coruja	11.98 ± 0.25 Ac	7.65 ± 0.28 Bf	159 ± 4.23 Bd	210 ± 3.61 Ab	0.076 ± 0.003 Ab	0.036 ± 0.001 Be
V14—Paulistinha	11.07 ± 0.42 Ac	9.61 ± 0.19 Be	199 ± 3.45 Ac	213 ± 4.97 Ab	0.056 ± 0.002 Ac	0.045 ± 0.002 Bd
V15—Sempre Verde	11.30 ± 0.06 Bc	17.06 ± 0.20 Aa	203 ± 4.72 Bc	227 ± 2.22 Aa	0.056 ± 0.001 Bc	0.075 ± 0.001 Ab

Equal uppercase letters in the rows and lowercase letters in the column do not differ by Student’s *t*-test and Scott–Knott test at 5% probability level, respectively.

**Table 3:** F-test and means test for stomatal conductance (*gs*) (mol (H_2_O) m^−2^ s^−1^), transpiration (*E*) (mmol (H_2_O) m^−2^ s^−1^), and instantaneous water use efficiency (*WUEi*) [(µmol (CO_2_) m^−2^ s^−1^) (mmol (H_2_O) m^−2^ s^−1^)^−1^] of traditional varieties of cowpea subjected to salinity levels of irrigation water.

F-Test						
Sources of Variation	*gs*		*E*		*WUEi*	
Block	0.000		0.000		0.000	
Salinity	0.031		0.000		0.000	
Varieties	0.000		0.000		0.000	
Salinity × Varieties	0.000		0.000		0.000	
Means comparison test (Standard Deviation, *n* = 5)						
Varieties	*gs*		*E*		*WUEi*	
	0.5 dS m^−1^	4.5 dS m^−1^	0.5 dS m^−1^	4.5 dS m^−1^	0.5 dS m^−1^	4.5 dS m^−1^
V1—Boquinha	0.21 ± 0.016 Aa	0.11 ± 0.004 Be	4.32 ± 0.19 Aa	1.98 ± 0.07 Be	3.94 ± 0.11 Bb	5.12 ± 0.38 Af
V2—Ceará	0.09 ± 0.001 Be	0.18 ± 0.006 Ab	2.47 ± 0.03 Bf	3.49 ± 0.11 Ab	3.30 ± 0.07 Ac	3.29 ± 0.19 Ah
V3—Costela de Vaca	0.14 ± 0.001 Ac	0.13 ± 0.004 Ac	3.48 ± 0.02 Ac	1.84 ± 0.04 Be	4.33 ± 0.12 Ba	8.22 ± 0.15 Ab
V4—Lisão	0.14 ± 0.002 Ac	0.14 ± 0.005 Ac	3.77 ± 0.03 Ab	2.57 ± 0.05 Bc	3.12 ± 0.03 Bc	4.78 ± 0.14 Af
V5—Canário	0.06 ± 0.002 Bg	0.11 ± 0.002 Ae	1.69 ± 0.07 Bh	2.47 ± 0.01 Ad	4.37 ± 0.23 Aa	4.51 ± 0.08 Ag
V6—Pingo de Ouro	0.08 ± 0.007 Bf	0.13 ± 0.001 Ac	2.28 ± 0.14 Ag	1.74 ± 0.01 Be	3.66 ± 0.12 Bb	7.50 ± 0.14 Ac
V7—Roxão	0.13 ± 0.007 Ac	0.09 ± 0.006 Bf	3.22 ± 0.12 Ad	1.64 ± 0.06 Bf	3.61 ± 0.11 Bb	5.96 ± 0.29 Ae
V8—Feijão Branco	0.15 ± 0.006 Ab	0.09 ± 0.004 Bf	3.83 ± 0.11 Ab	2.40 ± 0.09 Bd	2.82 ± 0.07 Bd	3.35 ± 0.14 Ah
V9—Canapu Branco	0.16 ± 0.006 Ab	0.11 ± 0.002 Be	3.63 ± 0.10 Ac	1.64 ± 0.02 Bf	2.74 ± 0.06 Bd	6.83 ± 0.28 Ad
V10—Canapu Miúdo	0.10 ± 0.002 Ae	0.03 ± 0.004 Bg	2.59 ± 0.02 Af	0.89 ± 0.09 Bg	4.05 ± 0.13 Aa	3.11 ± 0.20 Bh
V11—Ovo de Peru	0.12 ± 0.002 Ad	0.08 ± 0.002 Bf	2.83 ± 0.04 Ae	1.80 ± 0.02 Be	2.95 ± 0.04 Bd	4.45 ± 0.13 Ag
V12—Baeta	0.10 ± 0.007 Ae	0.11 ± 0.002 Ae	2.86 ± 0.17 Ae	1.44 ± 0.02 Bf	3.28 ± 0.15 Bc	9.44 ± 0.20 Aa
V13—Coruja	0.11 ± 0.002 Ad	0.09 ± 0.004 Bf	2.87 ± 0.05 Ae	2.42 ± 0.11 Bd	4.18 ± 0.08 Aa	3.17 ± 0.07 Bh
V14—Paulistinha	0.13 ± 0.007 Ac	0.12 ± 0.002 Bd	3.32 ± 0.13 Ad	2.69 ± 0.04 Bc	3.34 ± 0.06 Ac	3.58 ± 0.12 Ah
V15—Sempre Verde	0.14 ± 0.006 Bc	0.26 ± 0.005 Aa	3.37 ± 0.08 Bd	4.02 ± 0.05 Aa	3.36 ± 0.08 Bc	4.24 ± 0.04 Ag

Equal uppercase letters in the rows and lowercase letters in the column do not differ by Student’s *t*-test and Scott–Knott test at 5% probability level, respectively.

**Table 4:** F-test and means test for initial fluorescence (*Fo*) (µmol (photons) m^−2^ s^−1^), maximum fluorescence (*Fm*) (µmol (photons) m^−2^ s^−1^), variable fluorescence (*Fv*) (µmol (photons) m^−2^ s^−1^), the maximum quantum efficiency of photosystem II (*Fv/Fm*), the quantum efficiency of photosystem II (*Y(II)*), electron transport rate (*ETR*) (µmol (photons) m^−2^ s^−1^), minimum fluorescence of the illuminated plant tissue (Fo’) (µmol (photons) m^−2^ s^−1^), photochemical quenching coefficient (*qL*), the quantum yield of regulated photochemical quenching (*Y(NPQ)*), and the quantum yield of non-regulated photochemical quenching (*Y(NO)*) of traditional varieties of cowpea subjected to salinity levels of irrigation water.

F-Test (*p*-Value)					
Sources of Variation	*Fo*	*Fm*	*Fv*	*Fv/Fm*	*Y(II)*
Block	0.000	0.000	0.000	0.442	0.445
Salinity	0.037	0.235	0.046	0.003	0.010
Varieties	0.314	0.485	0.475	0.313	0.881
Salinity × Varieties	0.616	0.963	0.983	0.668	0.222
Means comparison test (Standard Deviation, *n* = 75)					
Salinity (dS m^−1^)	*Fo*	*Fm*	*Fv*	*Fv/Fm*	*Y(II)*
0.5	777.94 ± 11.21 A	3128.91 ± 35.94 A	2351.0 ± 33.99 B	0.75 ± 0.004 B	0.68 ± 0.008 A
4.5	749.48 ± 9.16 B	3178.00 ± 31.37 A	2428.5 ± 26.05 A	0.76 ± 0.002 A	0.65 ± 0.011 B
F-test (*p*-value)					
Sources of Variation	*ETR* ^1^	*Fo’* ^1^	*qL* ^1^	*Y(NPQ)* ^1^	*Y(NO)* ^1^
Block	0.000	0.2068	0.110	0.397	0.378
Salinity	0.000	0.9214	0.140	0.005	0.736
Varieties	0.308	0.5083	0.840	0.812	0.833
Salinity × Varieties	0.635	0.3448	0.626	0.203	0.393
Means comparison test (Standard Deviation, *n* = 75)					
Salinity (dS m^−1^)	*ETR*	*Fo’*	*qL*	*Y(NPQ)*	*Y(NO)*
0.5	29.66 ± 2.47 B	2.85 ± 0.102 A	0.014 ± 0.0005 A	0.27 ± 0.007 B	0.05 ± 0.0010 A
4.5	53.10 ± 4.07 A	2.82 ± 0.082 A	0.013 ± 0.0005 A	0.30 ± 0.010 A	0.05 ± 0.0012 A

^1^ Data transformed to square root. Equal uppercase letters in columns do not differ by Student’s *t*-test at a 5% probability level.

**Table 5:** F-test and means test for stem diameter (SD, mm) and the number of leaves (NL) of traditional varieties of cowpea subjected to salinity levels of irrigation water.

F-Test (*p*-Value)		
Sources of Variation	SD	NL
Block	0.871	0.035
Salinity	0.000	0.000
Varieties	0.000	0.000
Salinity × Varieties	0.159	0.329
Means comparison test (Standard Deviation, *n* = 10)		
Varieties	SD	NL
V1—Boquinha	10.1 ± 1.04 a	15.5 ± 3.36 c
V2—Ceará	9.6 ± 0.65 a	22.8 ± 3.77 a
V3—Costela de Vaca	7.9 ± 1.01 b	14.0 ± 3.19 c
V4—Lisão	8.2 ± 0.61 b	17.7 ± 3.45 b
V5—Canário	8.1 ± 0.41 b	16.9 ± 3.26 b
V6—Pingo de Ouro	8.1 ± 0.32 b	13.6 ± 2.35 c
V7—Roxão	7.9 ± 0.29 b	14.7 ± 2.11 c
V8—Feijão Branco	7.6 ± 0.54 b	11.4 ± 3.19 c
V9—Canapu Branco	8.0 ± 0.41 b	12.9 ± 2.10 c
V10—Canapu Miúdo	9.1 ± 0.58 a	16.7 ± 3.21 b
V11—Ovo de Peru	9.2 ± 0.44 a	18.9 ± 4.21 b
V12—Baeta	7.9 ± 0.29 b	11.4 ± 1.88 c
V13—Coruja	7.9 ± 0.46 b	17.7 ± 2.81 b
V14—Paulistinha	7.7 ± 0.57 b	13.5 ± 2.80 c
V15—Sempre Verde	8.1 ± 0.43 b	16.1 ± 3.86 b
Means comparison test (Standard Deviation, *n* = 75)		
Salinity	SD (mm)	NL
0.5 dS m^−1^	9.0 ± 0.15 A	20.8 ± 0.74 A
4.5 dS m^−1^	7.7 ± 0.14 B	10.4 ± 0.34 B

Equal uppercase letters in the rows and lowercase letters in the column do not differ by Student’s *t*-test and Scott–Knott test at 5% probability level, respectively.

**Table 6:** F-test and means test for main branch length (MBL, cm) and shoot dry mass (SDM, g) of traditional varieties of cowpea subjected to salinity levels of irrigation water.

F-Test (*p*-Value)				
Sources of Variation	MBL		SDM	
Block	0.277		0.040	
Salinity	0.000		0.000	
Varieties	0.000		0.002	
Salinity x Varieties	0.006		0.015	
Means comparison test (Standard Deviation, *n* = 5)				
Varieties	MBL		SDM	
	0.5 dS m^−1^	4.5 dS m^−1^	0.5 dS m^−1^	4.5 dS m^−1^
V1—Boquinha	202.4 ± 19.95 Ab	169.0 ± 16.79 Ab	26.9 ± 2.36 Ab	20.4 ± 2.00 Ba
V2—Ceará	253.8 ± 4.02 Aa	200.4 ± 9.21 Aa	32.8 ± 1.41 Aa	13.8 ± 1.67 Bb
V3—Costela de Vaca	266.4 ± 10.61 Aa	178.6 ± 26.79 Bb	31.7 ± 4.09 Aa	13.4 ± 2.50 Bb
V4—Lisão	193.4 ± 16.58 Ab	167.0 ± 27.82 Ab	23.3 ± 1.46 Ab	11.9 ± 1.70 Bb
V5—Canário	222.4 ± 22.46 Ab	222.2 ± 22.44 Aa	30.5 ± 0.91 Aa	17.4 ± 2.05 Ba
V6—Pingo de Ouro	242.8 ± 11.62 Aa	162.4 ± 18.52 Bb	24.5 ± 2.23 Ab	16.6 ± 2.59 Ba
V7—Roxão	276.0 ± 16.90 Aa	202.8 ± 29.67 Ba	25.8 ± 1.43 Ab	21.3 ± 1.80 Aa
V8—Feijão Branco	186.6 ± 14.18 Ab	167.0 ± 15.01 Ab	26.6 ± 1.28 Ab	18.3 ± 1.12 Ba
V9—Canapu Branco	297.4 ± 28.01 Aa	165.0 ± 19.02 Bb	23.2 ± 2.38 Ab	15.9 ± 2.10 Bb
V10—Canapu Miúdo	236.6 ± 14.94 Aa	148.0 ± 25.96 Bb	26.8 ± 1.85 Ab	13.4 ± 1.45 Bb
V11—Ovo de Peru	250.6 ± 14.32 Aa	247.8 ± 22.97 Aa	31.0 ± 4.26 Aa	21.3 ± 1.22 Ba
V12—Baeta	228.3 ± 8.22 Ab	82.8 ± 13.33 Bc	29.1 ± 1.28 Aa	17.9 ± 1.20 Ba
V13—Coruja	212.2 ± 11.62 Ab	168.0 ± 25.91 Ab	27.1 ± 1.73 Ab	16.1 ± 1.23 Bb
V14—Paulistinha	213.2 ± 19.62 Ab	164.6 ± 15.05 Ab	21.8 ± 1.43 Ab	11.6 ± 1.15 Bb
V15—Sempre Verde	238.3 ± 10.00 Aa	184.0 ± 36.33 Ab	32.9 ± 5.45 Aa	13.7 ± 3.01 Bb

Equal uppercase letters in the rows and lowercase letters in the column do not differ by Student’s *t*-test and Scott–Knott test at 5% probability level, respectively.

**Table 7:** F-test and means test for the number of pods per plant (*NPP*), the number of seeds per pod (*NSPo*), the number of seeds per plant (*NSPl*), and production per plant (*PP*, g) of traditional varieties of cowpea subjected to salinity levels of irrigation water.

F-Test (*p*-Value)				
Sources of Variation	NPP		NSPo	
Block	0.409		0.037	
Salinity	0.000		0.222	
Varieties	0.000		0.000	
Salinity × Varieties	0.005		0.009	
Means comparison test (Standard Deviation, *n* = 5)				
Varieties	NPP		NSPo	
	0.5 dS m^−1^	4.5 dS m^−1^	0.5 dS m^−1^	4.5 dS m^−1^
V1—Boquinha	6.8 ± 0.97 Ab	2.8 ± 0.58 Ba	11.6 ± 1.15 Bb	15.3 ± 0.48 Aa
V2—Ceará	4.8 ± 0.37 Ac	2.6 ± 0.68 Ba	13.4 ± 1.20 Aa	15.0 ± 0.61 Aa
V3—Costela de Vaca	4.2 ± 0.86 Ad	3.0 ± 0.55 Aa	9.9 ± 1.74 Ab	9.3 ± 0.79 Ab
V4—Lisão	4.8 ± 0.58 Ac	2.2 ± 0.58 Ba	11.9 ± 0.58 Ab	13.6 ± 0.84 Aa
V5—Canário	6.0 ± 0.55 Ac	2.4 ± 0.24 Ba	14.3 ± 1.34 Aa	10.0 ± 0.57 Bb
V6—Pingo de Ouro	5.2 ± 0.73 Ac	2.4 ± 0.24 Ba	13.9 ± 1.32 Aa	14.1 ± 0.62 Aa
V7—Roxão	6.2 ± 0.80 Ac	2.2 ± 0.49 Ba	13.5 ± 0.92 Aa	10.5 ± 1.50 Bb
V8—Feijão Branco	9.2 ± 1.46 Aa	3.6 ± 0.24 Ba	10.5 ± 1.03 Ab	10.1 ± 0.76 Ab
V9—Canapu Branco	6.0 ± 1.26 Ac	3.0 ± 1.76 Ba	13.9 ± 1.42 Aa	14.0 ± 1.66 Aa
V10—Canapu Miúdo	6.6 ± 0.51 Ab	3.0 ± 0.71 Ba	14.7 ± 0.94 Aa	12.6 ± 0.54 Aa
V11—Ovo de Peru	2.8 ± 0.37 Ad	2.4 ± 0.24 Aa	11.6 ± 1.13 Ab	9.9 ± 0.76 Ab
V12—Baeta	8.6 ± 0.24 Aa	3.6 ± 0.40 Ba	13.3 ± 1.10 Aa	12.9 ± 0.95 Aa
V13—Coruja	8.0 ± 0.84 Aa	4.0 ± 0.71 Ba	15.1 ± 0.85 Aa	12.1 ± 0.91 Ba
V14—Paulistinha	9.0 ± 0.01 Aa	2.8 ± 0.37 Ba	14.1 ± 1.10 Aa	13.0 ± 1.11 Aa
V15—Sempre Verde	3.6 ± 1.03 Ad	2.0 ± 0.32 Aa	11.8 ± 0.58 Ab	13.9 ± 1.08 Aa
F-test (*p*-value)				
Sources of Variation	NSPl		PP (g)	
Block	0.273		0.351	
Salinity	0.000		0.000	
Varieties	0.000		0.000	
Salinity x Varieties	0.000		0.003	
Means comparison test (Standard Deviation, *n* = 5)				
Varieties	NSPl		PP	
	0.5 dS m^−1^	4.5 dS m^−1^	0.5 dS m^−1^	4.5 dS m^−1^
V1—Boquinha	80.5 ± 14.41 Ab	42.3 ± 8.03 Ba	80.5 ± 0.90 Ab	42.3 ± 1.04 Ba
V2—Ceará	63.4 ± 5.90 Ac	38.5 ± 9.84 Aa	63.4 ± 0.97 Ac	38.5 ± 1.37 Aa
V3—Costela de Vaca	42.0 ± 13.61 Ac	26.8 ± 3.28 Aa	42.0 ± 2.17 Ac	26.8 ± 0.87 Aa
V4—Lisão	56.8 ± 6.65 Ac	28.3 ± 6.59 Ba	56.8 ± 2.09 Ac	28.3 ± 1.16 Ba
V5—Canário	83.7 ± 6.25 Ab	24.2 ± 3.07 Ba	83.7 ± 1.33 Ab	24.2 ± 0.54 Ba
V6—Pingo de Ouro	74.2 ± 15.67 Ab	33.6 ± 2.86 Ba	74.2 ± 1.50 Ab	33.6 ± 1.48 Ba
V7—Roxão	82.2 ± 9.74 Ab	23.9 ± 5.39 Ba	82.2 ± 1.20 Ab	23.9 ± 1.54 Ba
V8—Feijão Branco	91.4 ± 9.99 Ab	36.3 ± 3.36 Ba	91.4 ± 1.32 Ab	36.3 ± 0.22 Ba
V9—Canapu Branco	78.6 ± 14.86 Ab	36.3 ± 17.92 Ba	78.6 ± 1.86 Ab	36.3 ± 1.29 Ba
V10—Canapu Miúdo	96.8 ± 10.20 Ab	37.7 ± 8.78 Ba	96.8 ± 1.32 Ab	37.7 ± 1.17 Ba
V11—Ovo de Peru	30.8 ± 2.27 Ac	23.0 ± 0.95 Aa	30.8 ± 2.12 Ac	23.0 ± 0.34 Aa
V12—Baeta	113.8 ± 8.74 Aa	45.7 ± 3.88 Ba	113.8 ± 2.14 Aa	45.7 ± 0.55 Ba
V13—Coruja	121.0 ± 14.88 Aa	50.0 ± 11.61 Ba	121.0 ± 1.15 Aa	50.0 ± 1.67 Ba
V14—Paulistinha	126.6 ± 9.92 Aa	35.5 ± 3.99 Ba	126.6 ± 1.82 Aa	35.5 ± 0.88 Ba
V15—Sempre Verde	43.0 ± 12.79 Ac	28.0 ± 5.36 Aa	43.0 ± 2.46 Ac	28.0 ± 0.88 Aa

Equal uppercase letters in the rows and lowercase letters in the column do not differ by Student’s *t*-test and Scott–Knott test at 5% probability level, respectively.

**Table 8:** Chemical and physical analysis of the soil used in the experiment.

pH	OM	P	K^+^	Na^+^	Ca^2+^	Mg^2+^	Al^3+^	H + Al	SB	t	CEC	V	ESP
	(%)	-----(mg dm^−3^)----			------------------------- (cmol_c_ dm^−3^) ------------------							------%-----	
5.30	1.67	2.1	54.2	21.6	2.70	0.90	0.05	1.82	3.83	3.88	5.65	68	2.0
Density (kg dm^−3^)			Sand				Silt					Clay	
			--------------------------------------------- (g kg^−1^) ----------------------------------------										
1.60			820				30				150		

**Table 9:** Chemical characterization of Liqui-Plex Fruit^®^ foliar fertilizer.

Parameters								
N	Ca	S	B	Cu	Mn	Mo	Zn	OC
-------------------------------------------------g L^−1^-----------------------------------------------								%
73.50	14.70	78.63	14.17	0.74	73.50	1.47	73.50	2.45

N—nitrogen; Ca—calcium; S—sulfur; B—boron; Cu—copper; Mn—manganese; Mo—molybdenum; Zn—zinc; OC—organic carbon.

**Table 10:** Physical–chemical characterization of the water sources used in the experiment.

Water Sources	Parameters									
	pH	EC	K^+^	Na^+^	Mg^2+^	Ca^2+^	Cl^−^	CO_3_^2^^−^	HCO_3_^−^	SAR
		dS m^−1^	-----------------------------mmol_c_ L^−1^-------------------------------							
1	7.57	0.50	0.31	3.74	1.20	0.83	2.40	0.60	3.20	2.62
2	7.10	9.50	0.83	54.13	24.20	37.80	116.00	0.00	3.40	9.70

Water source 1—local-supply water; water source 2—reject brine; pH (H_2_O)—hydrogen potential in water; EC—electrical conductivity; K^+^—potassium; Na^+^—sodium; Mg^2+^—magnesium; Ca^2+^—calcium; Cl^−^—chlorine; CO_3_^2−^—carbonate; HCO_3_^−^—bicarbonate; SAR—sodium adsorption ratio, (mmol_c_ L^−1^) ^−0.5^.

## Data Availability

All other data are presented in the paper.
